# Temporal and spatial changes in wall shear stress during atherosclerotic plaque progression in mice

**DOI:** 10.1098/rsos.171447

**Published:** 2018-03-14

**Authors:** R. Xing, A. M. Moerman, Y. Ridwan, M. J. Daemen, A. F. W. van der Steen, F. J. H. Gijsen, K. van der Heiden

**Affiliations:** 1 Department of Biomedical Engineering, Thorax Center, Erasmus University Medical Center, Rotterdam, The Netherlands; 2 Department of Molecular Genetics, Erasmus University Medical Center, Rotterdam, The Netherlands; 3 Department of Pathology, Academic Medical Center, Amsterdam, The Netherlands

**Keywords:** atherosclerosis, plaque progression, contrast-enhanced micro-CT, wall shear stress, vulnerable plaque

## Abstract

Wall shear stress (WSS) is involved in atherosclerotic plaque initiation, yet its role in plaque progression remains unclear. We aimed to study (i) the temporal and spatial changes in WSS over a growing plaque and (ii) the correlation between WSS and plaque composition, using animal-specific data in an atherosclerotic mouse model. Tapered casts were placed around the right common carotid arteries (RCCA) of ApoE^−/−^ mice. At 5, 7 and 9 weeks after cast placement, RCCA geometry was reconstructed using contrast-enhanced micro-CT. Lumen narrowing was observed in all mice, indicating the progression of a lumen intruding plaque. Next, we determined the flow rate in the RCCA of each mouse using Doppler Ultrasound and computed WSS at all time points. Over time, as the plaque developed and further intruded into the lumen, absolute WSS significantly decreased. Finally at week 9, plaque composition was histologically characterized. The proximal part of the plaque was small and eccentric, exposed to relatively lower WSS. Close to the cast a larger and concentric plaque was present, exposed to relatively higher WSS. Lower WSS was significantly correlated to the accumulation of macrophages in the eccentric plaque. When pooling data of all animals, correlation between WSS and plaque composition was weak and no longer statistically significant. In conclusion, our data showed that in our mouse model absolute WSS strikingly decreased during disease progression, which was significantly correlated to plaque area and macrophage content. Besides, our study demonstrates the necessity to analyse individual animals and plaques when studying correlations between WSS and plaque composition.

## 1. Introduction

Wall shear stress (WSS) is the frictional force induced by blood flow acting on the endothelial lining of the vessel surface. WSS regulates the inflammatory status of the endothelium and thus determines the location of plaque development [1–3]. The progression of a plaque is a dynamic process, during which vessel geometry undergoes remodelling and narrowing, leading to substantial changes in the local WSS environment over time [4–7]. As the disease advances, plaques grow and they differ in composition, showing either a stable or vulnerable phenotype. Vulnerable plaques are characterized by the presence of a large necrotic core with a thin overlying fibrous cap and abundant infiltration of inflammatory cells [8]. Vulnerable plaques are prone to rupture and can cause subsequent thromboembolic events [9]. A substantial role for WSS in the initiation of atherosclerosis is established, however, whether WSS plays a role in plaque growth and/or progression remains under debate.

Evidence towards a role for WSS in plaque growth and progression does exist but is ambiguous. Clinical studies revealed that in carotid and coronary arteries, plaque rupture was predominantly located at the upstream shoulder of a plaque [10–13]. Plaque composition was heterogeneous along the direction of blood flow: thinner fibrous caps, larger necrotic cores and accumulation of macrophages were observed at the upstream shoulder of a plaque, correlating a vulnerable plaque phenotype to the location of rupture [12,14–17]. Several studies proposed that these regions were exposed to increased WSS, suggesting that high WSS may promote plaque vulnerability [18–21]. By contrast, low WSS was correlated to characteristics of plaque vulnerability in human and porcine coronary arteries [22–30]. Nevertheless, no direct association between evolution of WSS and plaque composition was established. We set out to study the dynamic process of disease progression and the concomitant spatial and temporal changes in lumen geometry and WSS using an atherosclerotic mouse model. In addition, we analysed the association between WSS and plaque composition.

We use an atherosclerotic animal model in which we can manipulate WSS *in vivo* and induce atherosclerotic plaque development [31–37]. We previously summarized the use of surgically manipulated flow models [38] and found that WSS distribution was studied only in some of these models and usually not taking full three-dimensional vessel geometry into account [34,36,39], while calculation of WSS critically depends on detailed vessel geometry and blood flow velocity [40]. In a previous cross-sectional study, using a tapered cast model [33], Pedrigi *et al.* [37] discovered that certain WSS metrics co-localized to the presence of a plaque with increased Oil Red O-lipid staining. However, quantitative analysis of plaque composition was not performed in this study and thus spatial correlations between WSS metrics and plaque composition could not be studied. The WSS metrics were investigated only at one time point while temporal data were lacking. Owing to the dynamic nature of plaque progression, monitoring WSS *in vivo* over time is a prerequisite. To the best of our knowledge, no studies have monitored temporal and spatial changes of WSS or correlated these to plaque composition in individual animals. We investigated the evolution of geometry, flow and WSS during plaque progression and the correlation between WSS and plaque composition in individual animals by analysing WSS *in vivo* at various time points during plaque progression. First, we obtained mouse-specific geometrical and flow data to compute WSS distribution at 5, 7 and 9 weeks after cast placement. This enabled us to monitor changes in WSS over the very same plaque during its growth and progression into an advanced plaque. At 9 weeks after cast placement, vessel samples were harvested and plaque composition was histologically characterized. Finally, regression analysis was performed to determine correlations between plaque composition and WSS.

## 2. Material and methods

### 2.1. Animals and cast placement

Female ApoE^−/−^ mice on C57BL/6 J background (*n* = 9) were purchased from Charles River (Maastricht, The Netherlands). At 13 weeks of age, normal chow diet was replaced with an atherogenic Western diet and provided ad libitum (Arie Blok, The Netherlands). Cast surgery was performed two weeks later on the animals under isoflurane-induced anaesthesia as described previously [33,41,42]. The average weight of the mice was 21.9 g. A tapering cast was placed around the right common carotid artery (RCCA), leading to changes in local WSS environment and subsequent plaque development. All animal experiments performed conform to the guidelines from Directive 2010/63/EU of the European Parliament on the protection of animals used for scientific purposes and approved by ethical committee of Erasmus MC Rotterdam.

### 2.2. Contrast-enhanced micro-CT imaging and right common carotid artery lumen reconstruction

**Figure 1.**
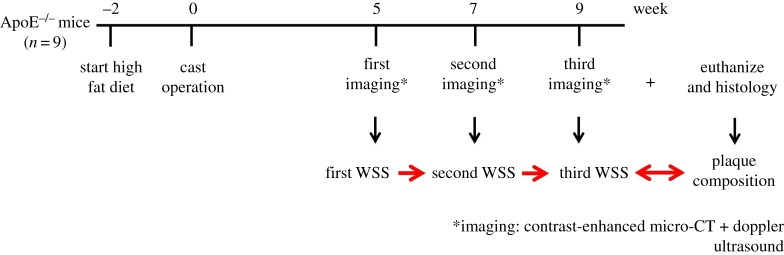
Experimental scheme. At *t* = −2 weeks, ApoE^−/−^ mice were fed a high fat diet; cast operation was performed on the right common carotid artery (RCCA) at *t* = 0 week to induce plaque growth (note that at *t* = 0, mice were 15 weeks of age); At *t* = 5, 7, 9 weeks during plaque progression, three-dimensional RCCA vessel geometry was reconstructed using contrast-enhanced micro-CT imaging. Blood flow velocity through the RCCA was measured by Doppler ultrasound; both micro-CT and ultrasound were carried out in each individual mouse; three-dimensional WSS maps at three time points of the same animal were thus generated using these imaging data. After the last imaging moment at *t* = 9 weeks, animals were euthanized, RCCA was excised for histological analyses.

To compute WSS in RCCA, we used time point and mouse-specific vessel geometries as the input, which was captured by contrast-enhanced micro-CT imaging (Quantum FX) with isotropic resolution of 40 µm. Images in Hounsfield unit (HU) were reconstructed. RCCA geometry of each animal at week 5, 7 and 9 was analysed using in-house developed modules in MeVisLab (MeVisLab 2.2.1) and Matlab (Matlab 2012). The segmentation protocol was previously established [40]. RCCA lumen surface was reconstructed from its origin at the bifurcation of the brachiocephalic artery, to its bifurcation into the internal and external carotid artery. Mice were anaesthetized with isoflurane with scanning parameters of 90 kvp, 160 µA, field of view 20 mm (figure 1). The radiation dose of each micro-CT scan was approximately 1.7 Gy. Mice underwent three scans in total, with 2-week time intervals. To determine a possible effect of radiation on atherosclerosis progression, we included a control group (*n* = 5) that was not imaged and analysed plaque composition 9 weeks after cast placement. No significant differences in plaque area or macrophage content were found (data not shown). Contrast agent eXIA 160 was used with an injection dose of 150 µl/25 grams of body weight. One mouse had paralysed hind limbs after the first imaging and was euthanized. One mouse was found dead before the third imaging at week 9. These two mice were excluded from subsequent analyses. Note that micro-CT is unable to capture outward remodelling of the vessel wall but allows visualization of lumen surface.

### 2.3. Doppler ultrasound imaging

Ultrasound imaging was performed using Vevo 2100 (VisualSonics) with a 40 MHz transducer. Blood velocity wave form was measured upstream of the RCCA under pulse-wave mode at five different time points: before and immediately after cast surgery, at 5, 7 and 9 weeks after cast surgery (figure 1). RCCA diameter was measured under M-mode at the same location as the pulse-wave measurement, thus allowing conversion of blood velocity (mm s^−1^) to flow (mm^3^ s^−1^) assuming a parabolic velocity profile.

### 2.4. Mesh and computational fluid dynamics simulations

RCCA lumen surface was smoothed using Vascular Modelling Tool Kit (VMTK 1.2). The superfluous ends at the proximal and distal side of the vessel surface were clipped and flow extensions were added. A volume mesh with prism layers was then generated using ICEM (ICEM-CFD 14.5, Ansys). Parameters including number of mesh elements, maximum element size, use of curvature/proximity based refinement, numbers and thickness of prism layers were optimized to obtain a mesh-independent solution resulting in approximately 640 000 elements. Surface area of the RCCA inlet was derived, enabling the calculation of location-specific blood flow velocity as boundary condition.

Several assumptions were made when solving the Navier–Stokes equations. First, the endothelial cells are exposed to time varying WSS levels. It is well established that the response of these cells is triggered by the time-averaged WSS that they are exposed to [43]. To obtain the time-averaged WSS, we can use steady flow simulations, using the average flow through the vessel as input [44]. In previous studies, it was demonstrated that other time-dependent WSS-derived parameters such as oscillatory shear index (OSI) are not relevant due to the geometry and flow conditions in this model [37,40], unlike in other vascular territories [45,46]. Furthermore, we assumed the WSS values are not influenced by the distensibility of the vessel wall. For blood flow in carotid arteries in mice, it was previously shown that distensibility is only important in the carotid bifurcation, and has a negligible effect in the common carotid artery [47]. Finally, blood was modelled as a Newtonian fluid, a valid assumption given the high shear rates in the carotid arteries of mice [48]. Therefore, we confined ourselves to steady flow simulation.

The resulting Navier–Stokes equations were solved by computational fluid dynamics (CFD) using Fluent (Fluent 14.5, Ansys). Blood was modelled to be incompressible. A constant viscosity of 3.5 × 10^−3^ kg m^−1^ s^−1^ and a density of 1060 kg m^−3^ were used [47,49]. Vessel wall was assumed to be rigid. A parabolic velocity profile was imposed as inlet boundary condition. Average blood flow velocity of the RCCA was derived from Doppler velocity measurements. For outlet boundary condition, zero pressure was used. WSS was then derived from the computed velocity field. Finally, post-processing and analysis were performed using CFD-Post (CFD-Post 14.5, Ansys) and Matlab.

### 2.5. Histological staining and analysis

**Figure 2.**
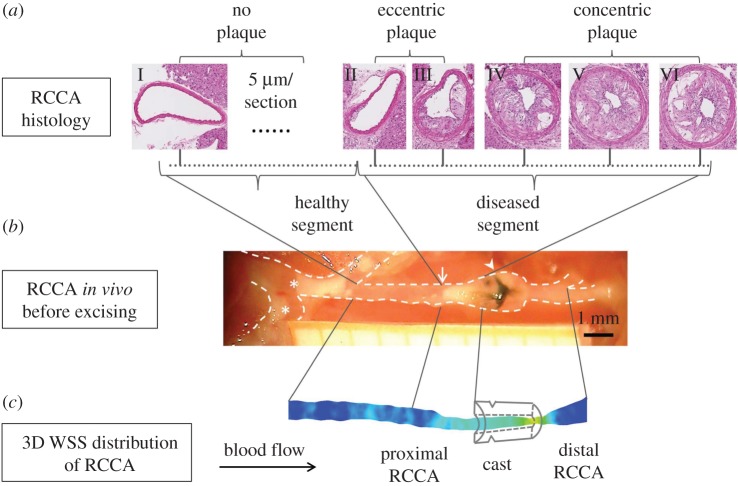
Spatial matching of histological sections to three-dimensional WSS map of an individual RCCA. (*a*) Representative H&E stained sections of the RCCA proximal to the cast, showing the healthy part, eccentric plaque part and the concentric plaque part. (*b*) Overview of the RCCA *in situ* before excision with 1 mm-spacing grid paper (arrowhead, black suture around the cast; arrow, proximal side of the plaque; asterisk, plaques located at the inner curve of the aortic arch and brachiocephalic artery). (*c*) Three-dimensional WSS map.

After final imaging at week 9 (figure 1), mice were euthanized by isoflurane overdose. The vascular system was then flushed with PBS through the left ventricle. Subsequently, 4% paraformaldehyde (PFA) in 0.15 mM PBS was used for pressure fixation at 100 mmHg [50]. Tissue around the RCCA was carefully cleaned, exposing the vasculature. The RCCA, cast, proximal brachiocephalic bifurcation and aortic arch could be clearly seen (figure 2*b*). Photos of RCCA were taken with a 1 mm grid strip. The cast was then carefully removed from the vessel. Proximally, the RCCA was excised 3 mm distal to the aortic arch and distally after the carotid bifurcation. The RCCA was then immersed in 4% PFA for 24 h at room temperature. Tissue samples were then processed and embedded in paraffin for histological analysis. We focused on the plaque upstream of the cast because its composition was relevant to our aim of study. We did not investigate the region within the cast because this region was exposed to increased WSS, and thus no plaque formation was observed. For the region downstream of the constriction, the cast was generally located close to the bifurcation. We can, therefore, expect a strong interplay between the presence of the jet downstream of the cast and the flow features associated with the division of the flow in the carotid bifurcation. These features will be greatly influenced by the flow division over the internal and external carotid artery. This ratio will change during the progression of the disease [47]. Since we did not measure flow in the internal or external carotid artery due to restricted time allocated for flow measurements, we cannot establish flow division and thus cannot evaluate WSS patterns downstream of the cast reliably. Therefore, we decided to exclude the plaque formed downstream in our analysis. Serial sections of the plaque upstream of the cast were collected. On average each plaque contains 21 serial sections (5 µm, at 50 µm interval), the two sections adjacent to the cast were excluded from the analysis to avoid possible confounding effects from the presence of the cast. Sections were stained for general plaque morphology (H&E), macrophages (CD68, Bio-Rad), endothelium (CD31, Dianova) and collagen (Resorcin-Fuchsine). Atherosclerotic plaque area, media area and relative macrophage area were quantified (BioPix iQ3.2). Necrotic core was defined as a-cellular, a-nuclear areas free of H&E staining [51].

### 2.6. Registration of histological staining to three-dimensional wall shear stress maps

For each animal, histological sections of the RCCA (figure 2*a*) were spatially registered to three-dimensional WSS maps at various time points (figure 2*c*) via the *in vivo* RCCA overview (figure 2*b*). We correlated plaque composition to WSS at different time points because although the effect of WSS on the endothelium is instant, the subsequent changes in plaque composition are not. Thus it is necessary to register WSS maps at week 5, 7 and 9 to plaque composition observed at the latest time point. To do the registration, we calculated RCCA vessel shrinkage from the *in vivo* situation to histology. Two landmarks are needed for this purpose. Since vessel segments with and without plaques were likely to exhibit different elasticity and therefore shrinkage, selection of landmarks should preferably be the beginning and the end of a plaque. The proximal edge of the plaque was clearly visible both on the RCCA overview (figure 2*b*, arrow) and histological sections (figure 2*a*, second section on the left). We thus selected it as the first landmark. The end of the plaque coincided with the proximal edge of the cast, which can be identified on histology. However, due to the scar tissue surrounding the cast, the distal edge of the plaque was not clearly visible on the RCCA overview. The black suture around the cast, however, can be easily spotted (figure 2*b*, arrowhead). Since the distance to the proximal edge of the cast was known (0.5 mm), we could pinpoint it as our second landmark. The average longitudinal shrinkage was 51% ± 20%, comparable to that reported previously [37]. On the three-dimensional WSS maps, location of the cast was identified from the micro-CT images. Using the individual shrinkage factor obtained for each mouse, histological sections were then spatially registered to the three-dimensional WSS maps at week 5, 7 and 9 (figure 2*c*). We investigated the association between plaque composition and WSS profiles at three time points as the duration between changes in WSS and the actual effect of that particular WSS on plaque composition is unknown.

According to the histological sections, proximal RCCA was divided into two segments: the healthy segment and the diseased segment. The corresponding segments were also identified on the three-dimensional RCCA geometry reconstructed from the micro-CT images at week 9. Since histology was only available at week 9 and micro-CT imaging can only capture lumen geometry but not plaque composition, it was not possible to pinpoint the border between the healthy and diseased segment on the three-dimensional RCCA geometry at week 5 and 7. We used the histology data obtained at week 9 to identify the healthy and diseased segments for these two time points. Lumen area in the proximal RCCA was derived from the three-dimensional RCCA geometry assuming circular cross-section [52]. Degree of stenosis was calculated as the percentage ratio between the minimum lumen area in the diseased segment and the average lumen area of the healthy segment. When analysing histological data, the diseased segment was further divided into two segments. Proximally, the plaque was composed of sections with eccentric plaque growth, in which plaque did not cover the entire circumferential direction. Close to the cast, the plaque covered the entire circumferential direction and was thus concentric (figure 2*a*).

### 2.7. Statistics

Data are presented as mean ± s.d. and analysed in Matlab and RStudio. Differences between samples were evaluated using a paired, two-tailed, Student's *t*-test or repeated-measures ANOVA. A Tukey's honest significant difference (HSD) *post hoc* test was performed after the ANOVA test. Correlations between histological quantifications and WSS data were performed by linear regression. A value of *p* < 0.05 was considered significant.

## 3. Results

### 3.1. *In vivo* micro-CT reveals various plaque progression profiles among animals

**Figure 3.**
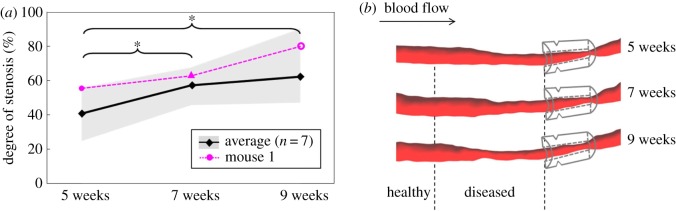
(*a*) Degree of stenosis of RCCA at 5, 7 and 9 weeks after cast placement: average of seven animals (black line, *p < 0.05, repeated-measures ANOVA); mouse 1 (pink line); shadow area indicates data range of seven animals. Notably, animals exhibited various plaque progression profiles. (*b*) Three-dimensional reconstruction of RCCA at 5, 7 and 9 weeks after cast placement of mouse 1. Direction of blood flow is indicated by the arrow in the upper left corner. Location of the cast is illustrated. Upstream to the cast, the RCCA is divided into a healthy segment and a diseased segment based on the week 9 histology data. Lumen narrowing can be observed over time, suggesting the development and progression of a lumen intruding plaque.

**Figure 4.**
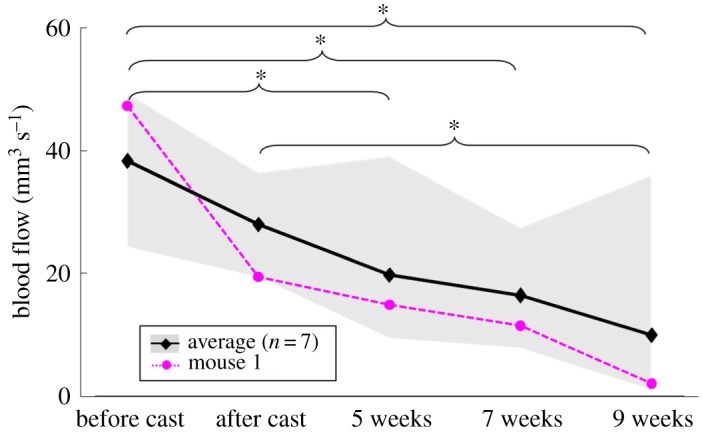
Blood flow along RCCA over time: average of seven animals showed a general decrease in blood flow over time (black line, * indicates *p* < 0.05, repeated-measures ANOVA); mouse 1 (pink line); shadow area indicates data range of all animals.

**Figure 5.**
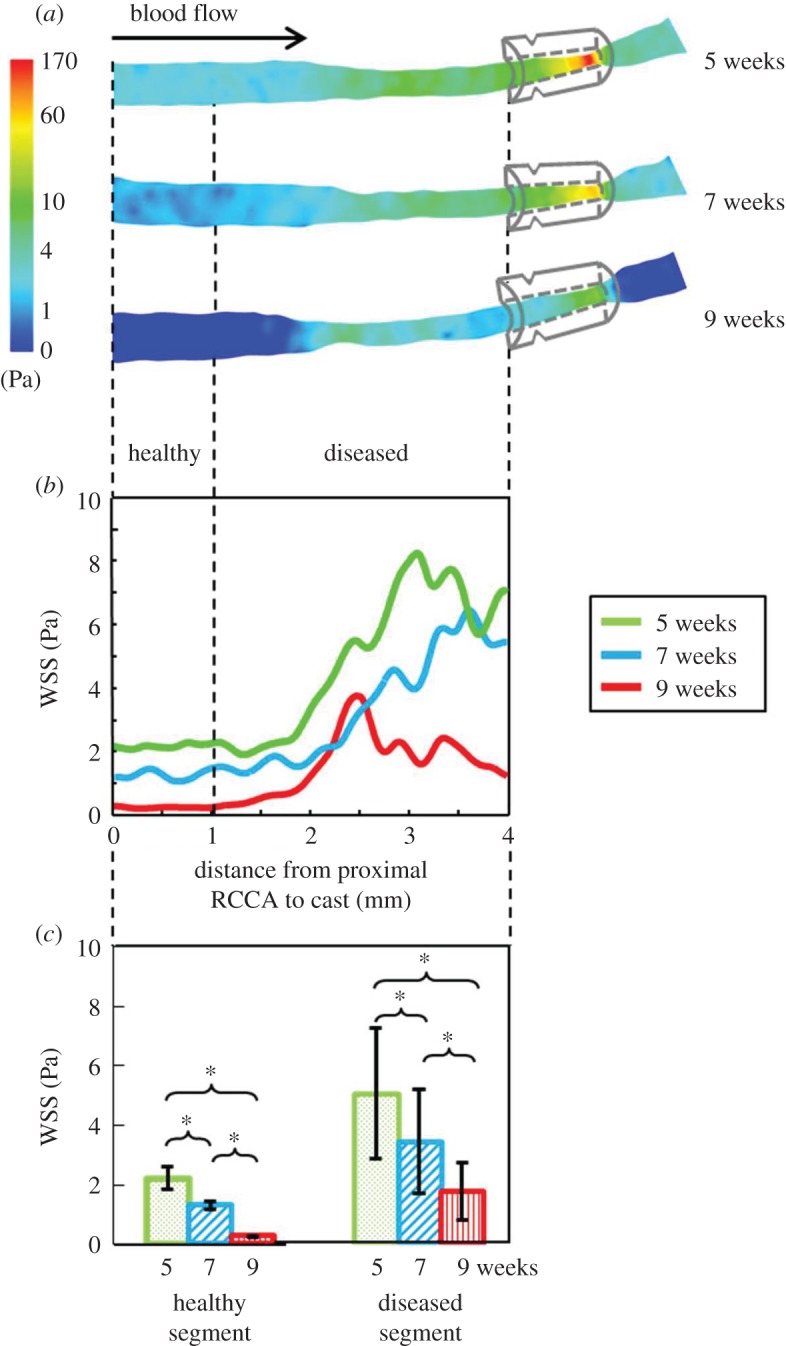
Illustration and quantification of WSS in a RCCA. (*a*) Three-dimensional WSS distribution in the RCCA of representative mouse 1 at 5, 7 and 9 weeks after cast placement. Circumferentially averaged WSS along the RCCA is shown (*b*). Proximally the RCCA was divided into a healthy and diseased segment, indicated by the vertical dashed line, based on the week 9 histology data. WSS distribution was heterogeneous over the diseased segment. Averaged WSS in both segments significantly decreased over time and was relatively higher in the diseased segment (Student's *t*-test, *p < 0.05).

**Figure 6.**
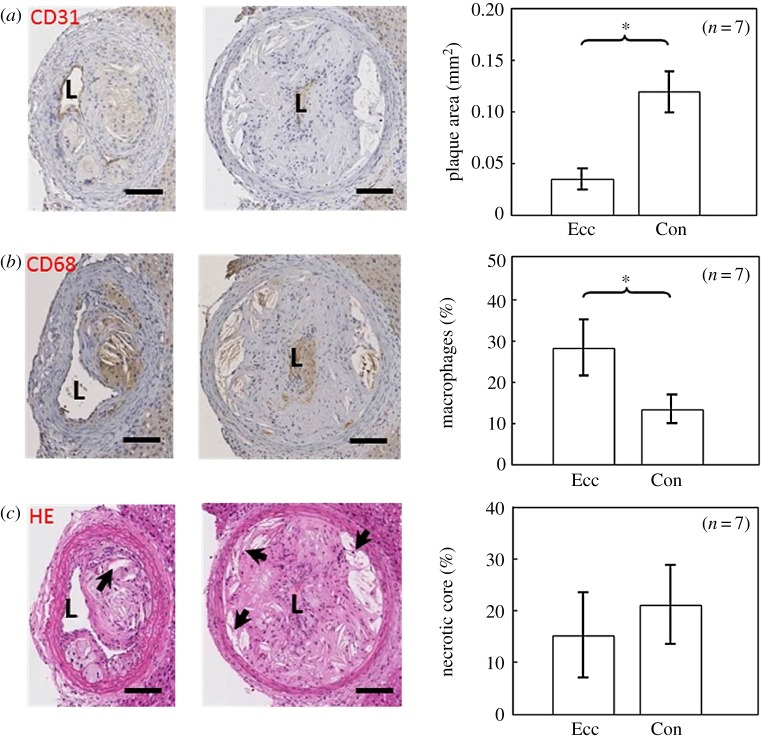
Characterization of plaque composition. Representative histological staining of the eccentric and concentric segment of representative mouse 1 and quantification of all mice is shown. (*a*) Plaque area (mm^2^) was delineated by CD31 endothelial staining; (*b*) relative macrophage area (%) was determined using CD68 staining; (*c*) relative necrotic core area (%) was delineated by H&E nuclei staining (arrows, necrotic cores); plaque area was significantly larger in the concentric segment, while macrophage area was larger in the eccentric segment (Student's *t*-test, *n* = 7, *p < 0.05). Scale bar is 100 µm.

Three-dimensional RCCA vessel geometries were reconstructed from contrast-enhanced micro-CT at 5, 7 and 9 weeks after cast placement. Various plaque progression profiles were evident among animals. Changes in lumen diameter were most evident between either 7 and 9 weeks, or 5 and 7 weeks. In a representative animal (mouse number 1 shown in figures 3–6), lumen narrowing was observed gradually over time (figure 3*a*, pink line, 3*b*). Degree of stenosis increased steadily from 55% at week 5 to 63% at week 7 and 80.0% at week 9 (figure 3*a*, pink line).

In all animals (*n* = 7), at week 5, lumen narrowing can be clearly seen in the diseased segment upstream of the cast (electronic supplementary material, figure S1), suggesting presence of a lumen intruding plaque. Degree of stenosis averaged among seven animals was 41 ± 11% at week 5, 57 ± 8% at week 7 (*p* < 0.05 versus week 5) and 62 ± 16% at week 9 (*p* < 0.05 versus week 5) (figure 3*a*, black line).

### 3.2. Blood flow in right common carotid artery decreased over time

Blood velocity was measured upstream of the RCCA before and immediately after cast surgery, and at 5, 7 and 9 weeks after cast placement. Blood flow in the RCCA was then converted using the measured parameters. In our representative animal, blood flow reduced from 47.3 to 19.4 mm^3^ s^−1^ after cast placement and further decreased during plaque progression, from 14.8 mm^3^ s^−1^ at week 5 to 11.4 mm^3^ s^−1^ at week 7 and 2.0 mm^3^ s^−1^ at week 9 (figure 4, pink line).

All mice except for one (mouse number 3 in electronic supplementary material, figure S1) showed a general decrease in blood flow over time. Blood flow in the RCCA averaged from all seven animals decreased immediately after cast placement from 38.4 ± 9.0 to 28.0 ± 5.5 mm^3^ s^−1^ (figure 4, black line). At weeks 5 and 7, blood flow further reduced to 19.8 ± 9.2 and 16.4 ± 6.0 mm^3^ s^−1^ (*p* < 0.05 versus before cast placement). Finally, at 9 weeks, blood flow was 9.9 ± 11.3 mm^3^ s^−1^, significantly lower than that before and after cast placement (*p* < 0.05).

### 3.3. Wall shear stress profiles as determined by animal-specific geometry and flow data

The spatial and temporal three-dimensional WSS distribution based on the mouse-specific geometry and flow data was computed at 5, 7 and 9 weeks after cast placement with CFD. Figure 5 illustrates the evolution of the three-dimensional WSS distribution of our representative animal. Before cast placement, average WSS was 14.4 ± 9.4 Pa decreasing to 12.9 ± 4.4 Pa after cast placement. At week 5, WSS was low upstream of the cast with an average value of 4.1 ± 2.2 Pa (figure 5*a*, upper panel). At week 7 and 9, as blood flow in the RCCA gradually reduced, WSS decreased in the RCCA (figure 5*a*, middle and lower panel). WSS distribution in the RCCA upstream of the cast was further analysed in the healthy and diseased segment. At week 5, the two segments were exposed to WSS of 2.2 ± 0.1 Pa and 5.0 ± 2.2 Pa respectively (figure 5*c*, green bars). Furthermore, WSS distribution was heterogeneous over the diseased segment (figure 5*b*, green line). Over time, as lumen area remained constant along the healthy segment, the decrease in blood flow led to lower WSS in this region (figure 5*b*, blue and red lines), with 1.3 ± 0.1 Pa at week 7 and 0.3 ± 0.0 Pa at week 9 (*p* < 0.05, figure 5*c*, blue bars). For the diseased segment, the lumen area narrowed gradually over time. Combined with the reduction in blood flow over time, WSS in this segment decreased significantly to 3.4 ± 1.7 Pa at week 7 and 1.7 ± 1.0 Pa at week 9 (*p* < 0.05, figure 5*c*, red bars).

Since various geometry features and blood flow patterns were observed among the seven mice, different WSS profiles were evident, presented in electronic supplementary material, figure S1. A general reduction in WSS over time was seen in five animals. Mouse number 2 showed an increase in WSS from 5 to 7 weeks, with a subsequent dramatic decrease from 7 to 9 weeks. Mouse number 3 was the only one showing a WSS increase over time, while in all other mice WSS was minimal in both the healthy and diseased segment at week 9. Furthermore, in all mice, the healthy segment was exposed to significantly lower levels of WSS when compared to the diseased segment at all time points.

### 3.4. Plaque morphology and composition varied within a single plaque

Histological analysis revealed the presence of one continuous plaque in the RCCA upstream of the cast 9 weeks after cast placement. However, differences in morphology and composition were found within this continuous plaque depending on the axial location. Histological sections of our representative animal are shown in figure 6. A smaller and eccentric plaque was observed proximally (figure 6, left section). Closer to the cast, the plaque was concentric and larger (figure 6, right section). Plaque area of the eccentric part was 0.04 mm^2^ compared to that of the concentric plaque 0.15 mm^2^. Relative macrophage area in the eccentric plaque was 24.6%, while in the concentric plaque a relative macrophage area of 13.9% was observed. In the eccentric plaque, relative necrotic core area was 2.7%, while it was 15.0% in the concentric plaque (data not shown).

The eccentric–concentric plaque morphology was present in all animals. Plaque area was significantly smaller in the eccentric plaque compared to that of the concentric plaque (0.03 ± 0.01 mm^2^ versus 0.12 ± 0.02 mm^2^, *p* < 0.05) (figure 6*a*). Macrophages were more concentrated in the eccentric plaque compared to that of the concentric plaque (figure 6*b*). On average, relative macrophage area in the eccentric plaque was 28.3 ± 6.9%, while in the concentric plaque a relative macrophage area of 13.4 ± 3.5% was observed (*p* < 0.05). In the eccentric plaque, relative necrotic core area appears to be somewhat smaller than that of the concentric plaque (figure 6*c*, 15.2 ± 8.2% versus 21.1 ± 7.7%). However, the difference between the eccentric and concentric plaque regarding relative necrotic core area was not statistically significant.

### 3.5. Correlation between wall shear stress and plaque composition

**Figure 7.**
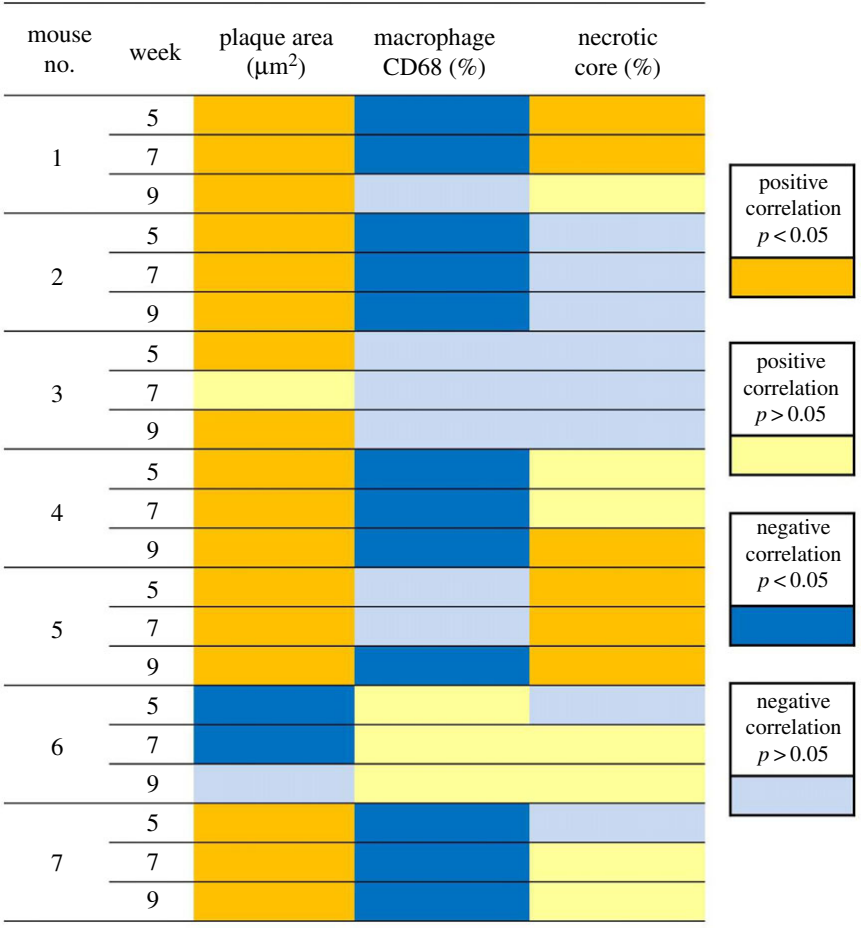
Correlation between WSS at all time points and plaque composition. Yellow, positive correlation, *p* < 0.05; light yellow, positive correlation, *p* > 0.05; blue, negative correlation, *p* < 0.05; light blue, negative correlation, *p* > 0.05.

Plaque area, macrophage content, and necrotic core area at week 9 was linked to WSS profiles at week 5, 7 and 9 (figure 7). In our representative animal, plaque area was positively associated with WSS and significant at all time points (*p* < 0.05). Accumulation of macrophages was negatively correlated to WSS and significant at both week 5 and 7 (*p* < 0.05), indicating that lower WSS suggested a pro-inflammatory profile in plaques. A positive correlation between relative necrotic core area and WSS level was positive at all three time points, however, only significant at week 5 and 7. Figure 7 summarizes the correlation between WSS and plaque area, macrophage content, and necrotic core area of all seven animals at three time points. Thus for each plaque parameter, there were 7 × 3 = 21 observations. In six out of seven animals at all time points, a positive and statistically significant correlation between WSS and plaque area was seen in 17 out of 21 observations. Inverse correlation between WSS and macrophages was observed in six out of seven animals where 12 out of 21 observations were statistically significant, and 6 out of 21 showed a non-significant negative correlation. The correlations between WSS and relative necrotic core size showed less significance. For relative necrotic core size, 6 out of 21 correlations were positive and statistically significant.

The correlation between plaque composition and WSS profiles were analysed in each individual animal. When histological data from all the animals was pooled together, as is usually the followed approach in these types of studies, a positive but very weak correlation between WSS and plaque area was seen, which was not statistically significant at any time point (the correlation coefficient was 0.08 at week 5, 0.1 at week 7 and 0.04 at week 9, *p* > 0.05), contrary to the result obtained from the individual animal analysis. Pooling data results in different and not necessarily correct correlation patterns.

## 4. Discussion

Our study described the temporal and spatial changes in vessel geometry, blood flow and concomitant WSS over an advanced murine plaque and its correlation to plaque area and macrophage content. First, we demonstrated that it was possible to follow changes in lumen area over time using contrast-enhanced micro-CT. Lumen narrowing was observed in all animals upstream of the cast, implying the formation of a lumen intruding plaque. The minimum degree of stenosis varied among animals at each time point, leading to different progression profiles over time. These differences in disease progression rate were not related to the initial degree of stenosis at week 5 or plaque size observed at week 9. Immediately after cast placement, flow reduction of 23.3 ± 19.8% was observed in the RCCA, comparable with previously reported values [33,37]. During plaque progression, as stenotic degree increased over time, blood flow in the RCCA decreased gradually from week 5 to 9. Flow decrease was also observed in animals with constant degree of stenosis over time. Therefore, the observed flow decrease was not only due to the increasing stenotic degree of the lumen intruding plaque, but also likely to be due to increasing resistance of the distal vascular bed beyond the carotid bifurcation. In general, blood flow decreased during plaque progression in all mice, except one. Our results strongly suggested that there were temporal and animal-specific variations in vessel geometry and blood flow and, therefore, one should not use generic values when computing WSS distribution as suggested previously [53].

Variations in the local geometrical and blood flow data at the time of investigation led to a complex spatial and temporal distribution of WSS *in vivo* among animals. As blood flow reduced and vessel diameter remained constant, RCCA was exposed to dramatically decreasing levels of *absolute* WSS over time. However, over all, WSS decreased over time and was minimal at week 9 in all animals but one. Generally it is believed that WSS over a lumen intruding plaque would increase over time due to the increased degree of stenosis. Our findings showed that this is not the case in this animal model, contrary to the general consensus [54]. Nevertheless, if we look at the spatial distribution of WSS in the upstream RCCA, *relative* WSS showed a gradual increase from the healthy segment to the eccentric and then concentric plaque segment, which is in line with the general consensus. Although relative WSS distribution followed the expected spatial pattern, one should keep in mind that it was on a background with dramatically decreased absolute WSS levels. This implies that previous studies which correlated high WSS to plaque pathogenesis using this mouse model should potentially be reinterpreted. This underlines again the importance of computing WSS on subject-specific flow and geometries.

**Figure 8.**
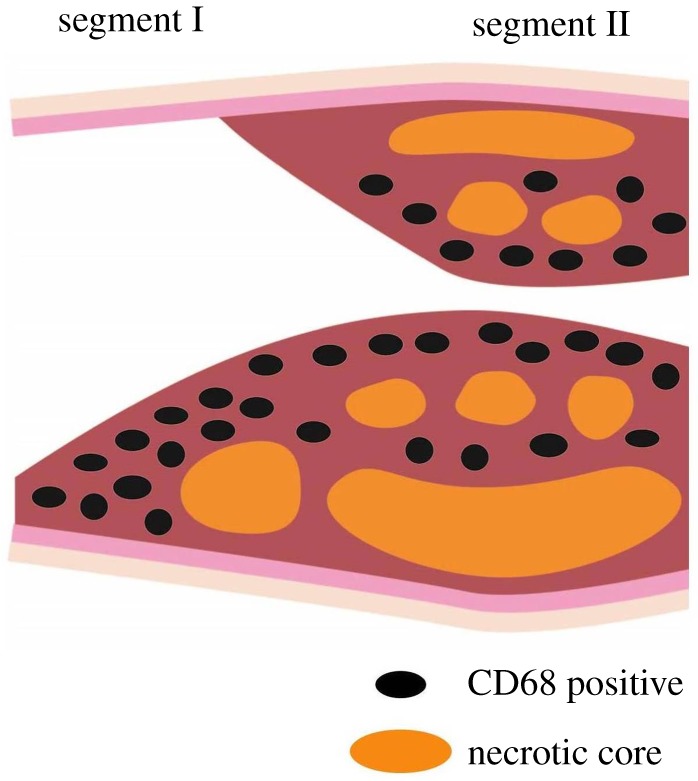
Schematic overview of plaque morphology. Proximally, the plaque was smaller and eccentric with abundant accumulation of macrophages. Closer to the cast, the plaque was larger and concentric.

To analyse correlations between WSS and plaque components, we analysed plaque composition after the final imaging moment. To visualize the spatial heterogeneity of plaque composition, we performed consistent sampling of the plaque, similar to the study reported before [55]. This analysis revealed the presence of one continuous plaque. Its composition varied in the axial direction (figure 8). In all animals, proximally, the plaque was small and eccentric, and abundant in macrophages. The macrophage concentration was comparable to that reported by Cheng *et al.* [33]. We observed that the plaque segment located closer to the cast was big and concentric. CT data demonstrated that vessel lumen narrowing was first observed close to the upstream part of the cast, suggesting that the plaque revealed here was present for a longer time and thus its composition was more advanced, while the proximal part of the plaque is ‘younger' and less advanced. The spatial heterogeneity of the plaque implied that sampling location should be taken into account when analysing plaque composition in any animal model for atherosclerosis or human atherosclerotic sample [15]. Results might be different if one only selects histological sections from either eccentric or concentric diseased segments.

Using mouse-specific *absolute WSS at a given time point* and using the histological data, we demonstrated a strong inverse relationship between macrophages and WSS. Macrophages accumulated in the proximal eccentric plaques where WSS was lowest, confirming the pro-inflammatory effect of low WSS reported before [1,14,56]. In this mouse model, the continuous exposure to reducing levels of WSS in the proximal eccentric plaque correlated to inflammation and thus plaque growth. However, regardless of the homogeneous distribution of WSS in the upstream RCCA, the proximal segment of the plaque had an eccentric composition. In addition, although the upstream part of the RCCA was homogeneously exposed to extremely low WSS, plaque was only present close to the cast. This evidence suggested that low WSS alone is not incentive enough to induce plaque initiation. We think that the plaque initially grew from the region close to the cast, and there might be factors (damage from the cast surgery, distribution of inflammatory cells or layout of fibrotic tissues) from the existing plaque that induced a non-homogeneous growth pattern in the proximal direction. In addition, we showed that six out of seven mice had a positive and significant correlation between WSS and plaque area. The one animal that showed contrasting correlations (mouse number 6 in figure 7) presented with such extensive plaque progression that the stenosis created by the plaque was more severe than the stenosis induced by cast placement (electronic supplementary material, figure S1).

In our study, we observed large variations in both WSS distribution and plaque composition among animals. Correlation between WSS distribution and plaque composition reduced when data were pooled. This implies that one can miss out on important correlations if the often-applied approach of pooling WSS and histological data is used. Similarly, De Wilde *et al.* [7] showed that pooling data led to misinterpretation of correlation between WSS metrics and plaque composition. Therefore, when analysing functional parameters from one to another, using subject-specific WSS and histological data is necessary.

## 5. Study limitations

A limitation of this study was that we were not able to perform rotational matching between the three-dimensional WSS maps to the lumen surface obtained from histology. Subdividing the three-dimensional WSS maps into cross-sections and matching it to the corresponding orientation of the histological sections would potentially increase information density. In our study, the upstream RCCA was a relatively straight segment where WSS distributed homogeneously along the lumen circumference. We thus expected little effect of performing rotational matching. In addition, we focused on computing WSS and did not compute wall stress which is expected to play a role, especially close to the cast [42], which is why we did not include the sections close to the cast in our histological analysis. A limitation of our model is that mice do not present with vulnerable plaques in accordance with the definition of a human vulnerable plaque; that is, mouse plaques are not rupture-prone [42]. The differences between mice—and animal models in general—and humans were reviewed by us and others [42,57,58]. The implication of these differences is that translation of the findings based on these animal data to patient studies should, therefore, be done cautiously. A relevant example of the difference between mice and humans pertains to cap thickness, a strong indicator of plaque vulnerability in human plaques, which is ill-defined in mice due to the presence of multiple necrotic cores, located deep in the plaque. We, therefore, decided not to put forward cap thickness analyses in this manuscript.

## 6. Conclusion

We quantified the temporal and spatial evolution of WSS in an atherosclerotic mouse model and we studied the correlation between the animal-specific WSS data and plaque composition in the same animal. Strikingly, due to the temporal changes in geometry and flow, WSS decreased over time during disease progression, contrary to the general belief. In the majority of the mice, lower WSS was associated with elevated levels of inflammation, which mainly localized proximally where the plaque was small and eccentric. Closer to the cast where a larger concentric plaque was observed, higher WSS was associated with increased plaque area. In addition, our study clearly demonstrated the necessity to analyse individual animals and plaques when studying correlations between WSS and plaque composition.

## Supplementary Material

3D WSS distribution in all mice.

## Data accessibility

The datasets supporting this article have been uploaded as part of the electronic supplementary material.

## References

[1] Malek AM, Alper SL, Izumo S 1999 Hemodynamic shear stress and its role in atherosclerosis. JAMA 282, 2035–2042. (doi:10.1001/jama.282.21.2035) 10591386 10.1001/jama.282.21.2035

[2] Chiu J-J, Usami S, Chien S 2009 Vascular endothelial responses to altered shear stress: pathologic implications for atherosclerosis. Ann. Med. 41, 19–28. (doi:10.1080/07853890802186921) 18608132 10.1080/07853890802186921

[3] Evans PC, Kwak BR 2013 Biomechanical factors in cardiovascular disease. Cardiovasc. Res. 99, 229–231. (doi:10.1093/cvr/cvt143) 23737494 10.1093/cvr/cvt143

[4] Slager CJ, Wentzel JJ, Gijsen FJH, Schuurbiers JCH, van der Wal AC, van der Steen AFW, Serruys PW 2005 The role of shear stress in the generation of rupture-prone vulnerable plaques. Nat. Clin. Pract. Cardiovasc. Med. 2, 401–407. (doi:10.1038/ncpcardio0274) 16119702 10.1038/ncpcardio0274

[5] Slager CJ, Wentzel JJ, Gijsen FJH, Thury A, van der Wal AC, Schaar JA, Serruys PW 2005 The role of shear stress in the destabilization of vulnerable plaques and related therapeutic implications. Nat. Clin. Pract. Cardiovasc. Med. 2, 456–464. (doi:10.1038/ncpcardio0298) 16265586 10.1038/ncpcardio0298

[6] Koskinas KC et al. 2010 Natural history of experimental coronary atherosclerosis and vascular remodeling in relation to endothelial shear stress: a serial, *in vivo* intravascular ultrasound study. Circulation 121, 2092–2101. (doi:10.1161/CIRCULATIONAHA.109.901678) 20439786 10.1161/CIRCULATIONAHA.109.901678 PMC2902864

[7] De Wilde D, Trachet B, De Meyer GRY, Segers P 2016 Shear stress metrics and their relation to atherosclerosis: an *in vivo* follow-up study in atherosclerotic mice. Ann. Biomed. Eng. 44, 2327–2338. (doi:10.1007/s10439-015-1540-z) 26695938 10.1007/s10439-015-1540-z

[8] Virmani R, Burke AP, Kolodgie FD, Farb A 2002 Vulnerable plaque: the pathology of unstable coronary lesions. J. Interv. Cardiol. 15, 439–446. (doi:10.1111/j.1540-8183.2002.tb01087.x) 12476646 10.1111/j.1540-8183.2002.tb01087.x

[9] Stary HC et al. 1995 A definition of advanced types of atherosclerotic lesions and a histological classification of atherosclerosis. A report from the Committee on Vascular Lesions of the Council on Arteriosclerosis, American Heart Association. Circulation 92, 1355–1374. (doi:10.1161/01.CIR.92.5.1355) 7648691 10.1161/01.cir.92.5.1355

[10] Fujii K et al. 2003 Intravascular ultrasound assessment of ulcerated ruptured plaques. Circulation 108, 2473–2478. (doi:10.1161/01.CIR.0000097121.95451.39) 14610010 10.1161/01.CIR.0000097121.95451.39

[11] de Weert TT, Cretier S, Groen HC, Homburg P, Cakir H, Wentzel JJ, Dippel DWJ, van der Lugt A 2009 Atherosclerotic plaque surface morphology in the carotid bifurcation assessed with multidetector computed tomography angiography. Stroke 40, 1334–1340. (doi:10.1161/STROKEAHA.108.538439) 19265048 10.1161/STROKEAHA.108.538439

[12] Cicha I, Wörner A, Urschel K, Beronov K, Goppelt-Struebe M, Verhoeven E, Daniel WG, Garlichs CD 2011 Carotid plaque vulnerability: a positive feedback between hemodynamic and biochemical mechanisms. Stroke 42, 3502–3510. (doi:10.1161/STROKEAHA.111.627265) 21998063 10.1161/STROKEAHA.111.627265

[13] Daemen MJ, Ferguson MS, Gijsen FJ, Hippe DS, Kooi ME, Demarco K, van der Wal AC, Yuan C, Hatsukami TS 2016 Carotid plaque fissure: an underestimated source of intraplaque hemorrhage. Atherosclerosis 254, 102–108. (doi:10.1016/j.atherosclerosis.2016.09.069) 27718372 10.1016/j.atherosclerosis.2016.09.069 PMC5533085

[14] Dirksen MT, van der Wal AC, van den Berg FM, van der Loos CM, Becker AE 1998 Distribution of inflammatory cells in atherosclerotic plaques relates to the direction of flow. Circulation 98, 2000–2003. (doi:10.1161/01.CIR.98.19.2000) 9808596 10.1161/01.cir.98.19.2000

[15] Fagerberg B, Ryndel M, Kjelldahl J, Akyürek LM, Rosengren L, Karlström L, Bergström G, Olson FJ 2010 Differences in lesion severity and cellular composition between *in vivo* assessed upstream and downstream sides of human symptomatic carotid atherosclerotic plaques. J. Vasc. Res. 47, 221–230. (doi:10.1159/000255965) 19893319 10.1159/000255965

[16] Nakazawa G, Yazdani SK, Finn AV, Vorpahl M, Kolodgie FD, Virmani R 2010 Pathological findings at bifurcation lesions: the impact of flow distribution on atherosclerosis and arterial healing after stent implantation. J. Am. Coll. Cardiol. 55, 1679–1687. (doi:10.1016/j.jacc.2010.01.021) 20394871 10.1016/j.jacc.2010.01.021

[17] Samady H, Eshtehardi P, McDaniel MC, Suo J, Dhawan SS, Maynard C, Timmins LH, Quyyumi AA, Giddens DP 2011 Coronary artery wall shear stress is associated with progression and transformation of atherosclerotic plaque and arterial remodeling in patients with coronary artery disease. Circulation 124, 779–788. (doi:10.1161/CIRCULATIONAHA.111.021824) 21788584 10.1161/CIRCULATIONAHA.111.021824

[18] Groen HC, Gijsen FJH, van der Lugt A, Ferguson MS, Hatsukami TS, van der Steen AFW, Yuan C, Wentzel JJ 2007 Plaque rupture in the carotid artery is localized at the high shear stress region: a case report. Stroke 38, 2379–2381. (doi:10.1161/STROKEAHA.107.484766) 17615365 10.1161/STROKEAHA.107.484766

[19] Fukumoto Y, Hiro T, Fujii T, Hashimoto G, Fujimura T, Yamada J, Okamura T, Matsuzaki M 2008 Localized elevation of shear stress is related to coronary plaque rupture: a 3-dimensional intravascular ultrasound study with *in-vivo* color mapping of shear stress distribution. J. Am. Coll. Cardiol. 51, 645–650. (doi:10.1016/j.jacc.2007.10.030) 18261684 10.1016/j.jacc.2007.10.030

[20] Yang C, Canton G, Yuan C, Ferguson M, Hatsukami TS, Tang D 2010 Advanced human carotid plaque progression correlates positively with flow shear stress using follow-up scan data: an in vivo MRI multi-patient 3D FSI study. J. Biomech. 43, 2530–2538. (doi:10.1016/j.jbiomech.2010.05.018) 20570268 10.1016/j.jbiomech.2010.05.018 PMC2937096

[21] Gijsen F, van der Giessen A, van der Steen A, Wentzel J 2013 Shear stress and advanced atherosclerosis in human coronary arteries. J. Biomech. 46, 240–247. (doi:10.1016/j.jbiomech.2012.11.006) 23261245 10.1016/j.jbiomech.2012.11.006

[22] Stone PH et al. 2003 Effect of endothelial shear stress on the progression of coronary artery disease, vascular remodeling, and in-stent restenosis in humans: *in vivo* 6-month follow-up study. Circulation 108, 438–444. (doi:10.1161/01.CIR.0000080882.35274.AD) 12860915 10.1161/01.CIR.0000080882.35274.AD

[23] Chatzizisis YS et al. 2008 Prediction of the localization of high-risk coronary atherosclerotic plaques on the basis of low endothelial shear stress: an intravascular ultrasound and histopathology natural history study. Circulation 117, 993–1002. (doi:10.1161/CIRCULATIONAHA.107.695254) 18250270 10.1161/CIRCULATIONAHA.107.695254

[24] Wentzel JJ, Chatzizisis YS, Gijsen FJH, Giannoglou GD, Feldman CL, Stone PH 2012 Endothelial shear stress in the evolution of coronary atherosclerotic plaque and vascular remodelling: current understanding and remaining questions. Cardiovasc. Res. 96, 234–243. (doi:10.1093/cvr/cvs217) 22752349 10.1093/cvr/cvs217

[25] Eshtehardi P et al. 2012 Association of coronary wall shear stress with atherosclerotic plaque burden, composition, and distribution in patients with coronary artery disease. J. Am. Heart Assoc. 1, e002543 (doi:10.1161/JAHA.112.002543) 23130168 10.1161/JAHA.112.002543 PMC3487351

[26] Koskinas KC et al. 2013 Thin-capped atheromata with reduced collagen content in pigs develop in coronary arterial regions exposed to persistently low endothelial shear stress significance. Arterioscler. Thromb. Vasc. Biol. 33, 1494–1504. (doi:10.1161/ATVBAHA.112.300827) 23640495 10.1161/ATVBAHA.112.300827 PMC3954496

[27] Vergallo R et al. 2014 Endothelial shear stress and coronary plaque characteristics in humans: combined frequency-domain optical coherence tomography and computational fluid dynamics study. Circ. Cardiovasc. Imaging 7, 905–911. (doi:10.1161/CIRCIMAGING.114.001932) 25190591 10.1161/CIRCIMAGING.114.001932

[28] Papafaklis MI et al. 2015 Effect of the local hemodynamic environment on the de novo development and progression of eccentric coronary atherosclerosis in humans: insights from PREDICTION. Atherosclerosis 240, 205–211. (doi:10.1016/j.atherosclerosis.2015.03.017) 25801012 10.1016/j.atherosclerosis.2015.03.017

[29] Han D et al. 2016 Relationship between endothelial wall shear stress and high-risk atherosclerotic plaque characteristics for identification of coronary lesions that cause ischemia: a direct comparison with fractional flow reserve. J. Am. Heart Assoc. 5, e004186 (doi:10.1161/JAHA.116.004186) 27993831 10.1161/JAHA.116.004186 PMC5210401

[30] Timmins LH, Molony DS, Eshtehardi P, McDaniel MC, Oshinski JN, Giddens DP, Samady H 2017 Oscillatory wall shear stress is a dominant flow characteristic affecting lesion progression patterns and plaque vulnerability in patients with coronary artery disease. J. R. Soc. Interface 14, 20160972 (doi:10.1098/rsif.2016.0972) 28148771 10.1098/rsif.2016.0972 PMC5332583

[31] Kumar A, Lindner V 1997 Remodeling with neointima formation in the mouse carotid artery after cessation of blood flow. Arterioscler. Thromb. Vasc. Biol. 17, 2238–2244. (doi:10.1161/01.ATV.17.10.2238) 9351395 10.1161/01.atv.17.10.2238

[32] Castier Y, Brandes RP, Leseche G, Tedgui A, Lehoux S 2005 p47phox-dependent NADPH oxidase regulates flow-induced vascular remodeling. Circ. Res. 97, 533–540. (doi:10.1161/01.RES.0000181759.63239.21) 16109921 10.1161/01.RES.0000181759.63239.21

[33] Cheng C, Tempel D, van Haperen R, van der Baan A, Grosveld F, Daemen MJAP, Krams R, de Crom R 2006 Atherosclerotic lesion size and vulnerability are determined by patterns of fluid shear stress. Circulation 113, 2744–2753. (doi:10.1161/CIRCULATIONAHA.105.590018) 16754802 10.1161/CIRCULATIONAHA.105.590018

[34] Nam D, Ni C, Rezvan A, Suo J, Budzyn K, Llanos A, Harrison D, Giddens D, Jo H 2009 Partial carotid ligation is a model of acutely induced disturbed flow, leading to rapid endothelial dysfunction and atherosclerosis. Am. J. Physiol. 297, 1535–1543. (doi:10.1152/ajpheart.00510.2009) 10.1152/ajpheart.00510.2009 PMC2770764 19684185

[35] Thim T et al. 2012 Wall shear stress and local plaque development in stenosed carotid arteries of hypercholesterolemic minipigs. J. Cardiovasc. Dis. Res. 3, 76–83. (doi:10.4103/0975-3583.95358) 22629022 10.4103/0975-3583.95358 PMC3354474

[36] Chen Y-C et al. 2013 A novel mouse model of atherosclerotic plaque instability for drug testing and mechanistic/therapeutic discoveries using gene and microRNA expression profiling. Circ. Res. 113, 252–265. (doi:10.1161/CIRCRESAHA.113.301562) 23748430 10.1161/CIRCRESAHA.113.301562

[37] Pedrigi RM et al. 2016 Influence of shear stress magnitude and direction on atherosclerotic plaque composition. R. Soc. open sci. 3, 160588 (doi:10.1098/rsos.160588) 27853578 10.1098/rsos.160588 PMC5099003

[38] Winkel LC, Hoogendoorn A, Xing R, Wentzel JJ, Van der Heiden K 2015 Animal models of surgically manipulated flow velocities to study shear stress-induced atherosclerosis. Atherosclerosis 241, 100–110. (doi:10.1016/j.atherosclerosis.2015.04.796) 25969893 10.1016/j.atherosclerosis.2015.04.796

[39] Cheng C et al. 2004 The role of shear stress in atherosclerosis: action through gene expression and inflammation? Cell Biochem. Biophys. 41, 279–294. (doi:10.1385/CBB:41:2:279) 15475614 10.1385/cbb:41:2:279

[40] Xing R, De Wilde D, McCann G, Ridwan Y, Schrauwen JTC, van der Steen AFW, Gijsen FJH, van der Heiden K 2016 Contrast-enhanced micro-CT imaging in murine carotid arteries: a new protocol for computing wall shear stress. Biomed. Eng. Online 15, 621–633. (doi:10.1186/s12938-016-0270-2) 10.1186/s12938-016-0270-2 PMC5259814 28155699

[41] Winkel LCJ, Groen HC, van Thiel BS, Müller C, van der Steen AFW, Wentzel JJ, de Jong M, Van der Heiden K 2014 Folate receptor–targeted single-photon emission computed tomography/computed tomography to detect activated macrophages in atherosclerosis: can it distinguish vulnerable from stable atherosclerotic plaques? Mol. Imaging 13, 61 (doi:10.2310/7290.2013.00061) 10.2310/7290.2013.00061 24757762

[42] van der Heiden K, Hoogendoorn A, Daemen MJ, Gijsen FJH 2015 Animal models for plaque rupture: a biomechanical assessment. Thromb. Haemost. 115, 501–508. (doi:10.1160/TH15-07-0614) 26607378 10.1160/TH15-07-0614

[43] Davies PF 2009 Hemodynamic shear stress and the endothelium in cardiovascular pathophysiology. Nat. Clin. Pract. Cardiovasc. Med. 6, 16–26. (doi:10.1038/ncpcardio1397) 19029993 10.1038/ncpcardio1397 PMC2851404

[44] Schrauwen JTC, Karanasos A, van Ditzhuijzen NS, Aben J-P, van der Steen AFW, Wentzel JJ, Gijsen FJH 2015 Influence of the accuracy of angiography-based reconstructions on velocity and wall shear stress computations in coronary bifurcations: a phantom study. PLoS ONE 10, e0145114 (doi:10.1371/journal.pone.0145114) 26690897 10.1371/journal.pone.0145114 PMC4686962

[45] Peiffer V, Sherwin SJ, Weinberg PD 2013 Does low and oscillatory wall shear stress correlate spatially with early atherosclerosis? A systematic review. Cardiovasc. Res. 99, 242–250. (doi:10.1093/cvr/cvt044) 23459102 10.1093/cvr/cvt044 PMC3695746

[46] Mohamied Y, Rowland EM, Bailey EL, Sherwin SJ, Schwartz MA, Weinberg PD 2015 Change of direction in the biomechanics of atherosclerosis. Ann. Biomed. Eng. 43, 16–25. (doi:10.1007/s10439-014-1095-4) 25138165 10.1007/s10439-014-1095-4 PMC4286626

[47] De Wilde D, Trachet B, Debusschere N, Iannaccone F, Swillens A, Degroote J, Vierendeels J, De Meyer GRY, Segers P 2015 Assessment of shear stress related parameters in the carotid bifurcation using mouse-specific FSI simulations. J. Biomech. 49, 2135–2142. (doi:10.1016/j.jbiomech.2015.11.048) 26655592 10.1016/j.jbiomech.2015.11.048

[48] Gijsen FJH, Allanic E, van de Vosse FN, Janssen JD 1999 The influence of the non-Newtonian properties of blood on the flow in large arteries: unsteady flow in a 90° curved tube. J. Biomech. 32, 705–713. (doi:10.1016/S0021-9290(99)00014-7) 10400358 10.1016/s0021-9290(99)00014-7

[49] Trachet B, Renard M, De Santis G, Staelens S, De Backer J, Antiga L, Loeys B, Segers P 2011 An integrated framework to quantitatively link mouse-specific hemodynamics to aneurysm formation in angiotensin II-infused ApoE^−/−^ mice. Ann. Biomed. Eng. 39, 2430–2444. (doi:10.1007/s10439-011-0330-5) 21614649 10.1007/s10439-011-0330-5

[50] Gage GJ, Kipke DR, Shain W 2012 Whole animal perfusion fixation for rodents. J. Vis. Exp. 30, 3564 (doi:10.3791/3564) 10.3791/3564 PMC3476408 22871843

[51] Seimon TA et al. 2009 Macrophage deficiency of p38alpha MAPK promotes apoptosis and plaque necrosis in advanced atherosclerotic lesions in mice. J. Clin. Invest. 119, 886–898. (doi:10.1172/JCI37262) 19287091 10.1172/JCI37262 PMC2662559

[52] Schürmann C, Gremse F, Jo H, Kiessling F, Brandes RP 2015 Micro-CT technique is well suited for documentation of remodeling processes in murine carotid arteries. PLoS ONE 10, e0130374 (doi:10.1371/journal.pone.0130374) 26086218 10.1371/journal.pone.0130374 PMC4472757

[53] Van Doormaal M, Zhou Y-Q, Zhang X, Steinman DA, Mark Henkelman R 2014 Inputs for subject-specific computational fluid dynamics simulation of blood flow in the mouse aorta. J. Biomech. Eng. 136, 101008 (doi:10.1115/1.4028104) 25070260 10.1115/1.4028104

[54] Wang Y et al. 2016 High shear stress induces atherosclerotic vulnerable plaque formation through angiogenesis. Regen. Biomater. 3, 257–267. (doi:10.1093/rb/rbw021) 27482467 10.1093/rb/rbw021 PMC4966293

[55] Pedrigi RM, de Silva R, Bovens SM, Mehta VV, Petretto E, Krams R 2014 Thin-cap fibroatheroma rupture is associated with a fine interplay of shear and wall stress. Arterioscler. Thromb. Vasc. Biol. 34, 2224–2231. (doi:10.1161/ATVBAHA.114.303426) 25060797 10.1161/ATVBAHA.114.303426

[56] VanderLaan PA, Reardon CA, Getz GS 2004 Site specificity of atherosclerosis: site-selective responses to atherosclerotic modulators. Arterioscler. Thromb. Vasc. Biol. 24, 12–22. (doi:10.1161/01.ATV.0000105054.43931.f0) 14604830 10.1161/01.ATV.0000105054.43931.f0

[57] Pedrigi RM et al. 2015 Inducing persistent flow disturbances accelerates atherogenesis and promotes thin cap fibroatheroma development in D374Y-PCSK9 hypercholesterolemic minipigs. Circulation 132, 1003–1012. (doi:10.1161/CIRCULATIONAHA.115.016270) 26179404 10.1161/CIRCULATIONAHA.115.016270

[58] Getz GS, Reardon CA 2012 Animal models of atherosclerosis. Arterioscler. Thromb. Vasc. Biol. 32, 1104–1115. (doi:10.1161/ATVBAHA.111.237693) 22383700 10.1161/ATVBAHA.111.237693 PMC3331926

