# Supplementary figures and images for: Temporal and spatial changes in wall shear stress during atherosclerotic plaque progression in mice
Source: R Soc Open Sci. 2018 Mar 14;5(3):171447. doi: 10.1098/rsos.171447 (PMC5882682; doi:10.1098/rsos.171447)

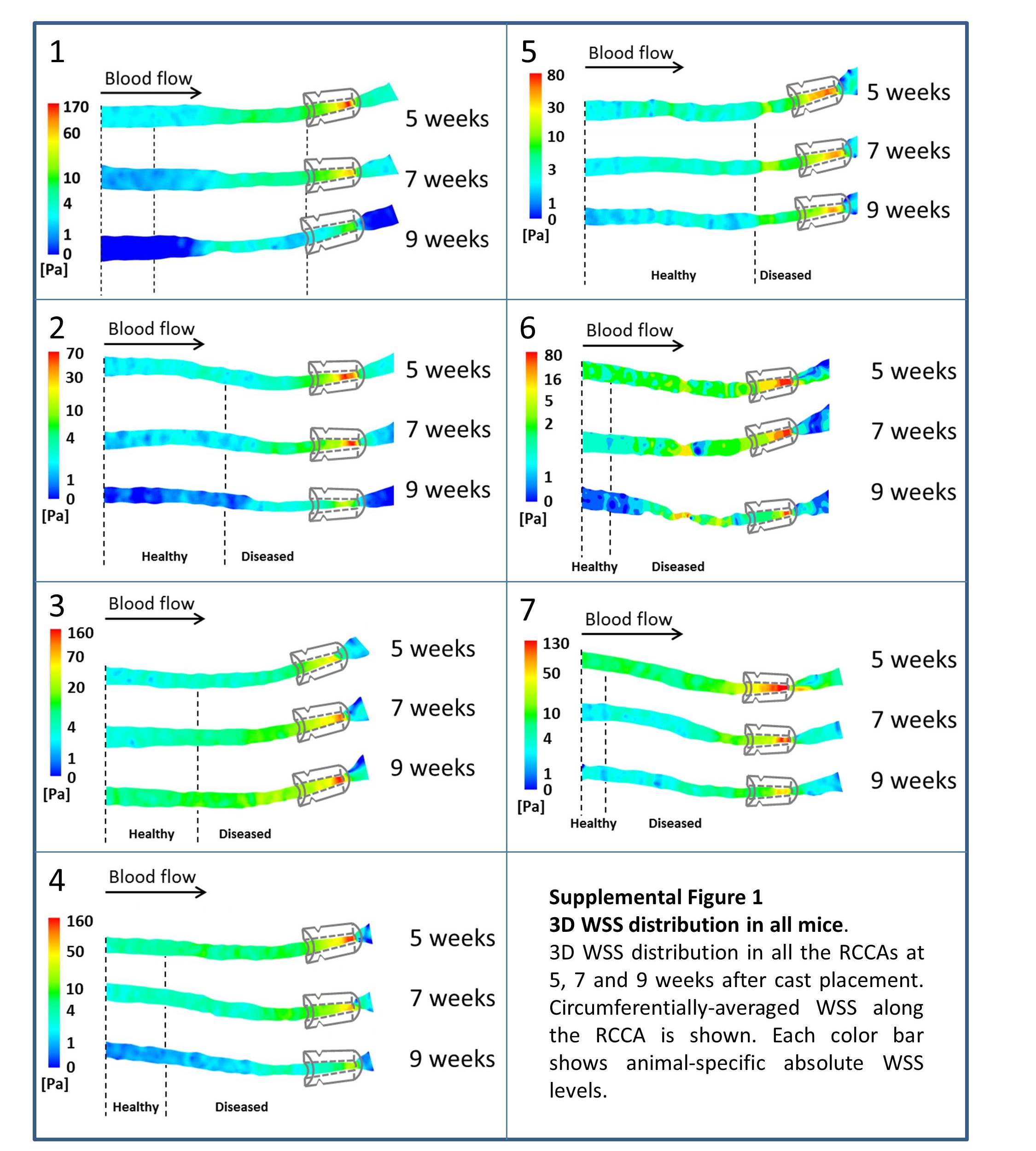

Supplement: 3D WSS distribution in all mice. [file rsos171447supp1.jpg]
